# Osteoprotegerin in Bone Metastases: Mathematical Solution to the Puzzle

**DOI:** 10.1371/journal.pcbi.1002703

**Published:** 2012-10-18

**Authors:** Marc D. Ryser, Yiding Qu, Svetlana V. Komarova

**Affiliations:** 1 Department of Mathematics and Statistics, McGill University, Montréal, Québec, Canada; 2 Department of Anatomy and Cell Biology, McGill University, Montréal, Québec, Canada; 3 Faculty of Dentistry, and Department of Anatomy and Cell Biology, McGill University, Montréal, Québec, Canada; University of Notre Dame, United States of America

## Abstract

Bone is a common site for cancer metastasis. To create space for their growth, cancer cells stimulate bone resorbing osteoclasts. Cytokine RANKL is a key osteoclast activator, while osteoprotegerin (OPG) is a RANKL decoy receptor and an inhibitor of osteoclastogenesis. Consistently, systemic application of OPG decreases metastatic tumor burden in bone. However, OPG produced locally by cancer cells was shown to enhance osteolysis and tumor growth. We propose that OPG produced by cancer cells causes a local reduction in RANKL levels, inducing a steeper RANKL gradient away from the tumor and towards the bone tissue, resulting in faster resorption and tumor expansion. We tested this hypothesis using a mathematical model of nonlinear partial differential equations describing the spatial dynamics of OPG, RANKL, PTHrP, osteoclasts, tumor and bone mass. We demonstrate that at lower expression rates, tumor-derived OPG enhances the chemotactic RANKL gradient and osteolysis, whereas at higher expression rates OPG broadly inhibits RANKL and decreases osteolysis and tumor burden. Moreover, tumor expression of a soluble mediator inducing RANKL in the host tissue, such as PTHrP, is important for correct orientation of the RANKL gradient. A meta-analysis of OPG, RANKL and PTHrP expression in normal prostate, carcinoma and metastatic tissues demonstrated an increase in expression of OPG, but not RANKL, in metastatic prostate cancer, and positive correlation between OPG and PTHrP in metastatic prostate cancer. The proposed mechanism highlights the importance of the spatial distribution of receptors, decoys and ligands, and can be applied to other systems involving regulation of spatially anisotropic processes.

## Author Summary

Breast and prostate cancers commonly metastasize to bone. To create more space for their expansion, metastatic tumors activate osteoclasts, the only cells capable of bone destruction. The main osteoclast stimulator is the cytokine RANKL, while osteoprotegerin (OPG) acts as a RANKL inhibitor. Systemic application of OPG leads to a decrease in tumor-associated bone destruction, but surprisingly, OPG produced locally by metastasizing cancer cells can enhance bone destruction and tumor growth. Here, we provide a novel explanation for these apparently contradictory experimental results: the osteolysis-promoting effect of OPG is due to a local reduction in RANKL levels, resulting in a spatial RANKL gradient oriented from tumor towards bone tissue. At low rates of OPG expression by cancer cells, such gradients result in the correct orientation of osteoclast movement and intensified bone resorption. We positively test our hypothesis by means of a partial differential equations model, and further substantiate our results with a meta-analysis of gene expression. Even though developed for the specific problem of bone metastases, our model naturally applies to other systems operating within a geometrically anisotropic environment.

## Introduction

Primary cancers develop metastatic tumors in distant sites and tissues of the body, and frequently, fatal outcome is due to those secondary rather than the primary tumors [1]. Bone is a common site for metastases and up to 70% of breast and prostate cancer patients develop secondary tumors in the bone environment [2]. While bone metastases are often classified as either osteolytic or osteoblastic, most metastases exhibit both components [1].

**Figure 1:**
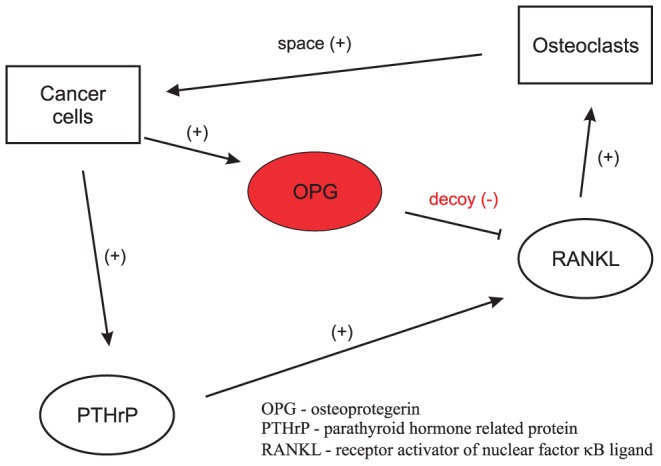
Interactions taken into account in the study. Two cell types are considered: cancer cells and osteoclasts. Osteoclasts positively affect cancer cells by providing space for tumor growth. Parathyroid hormone-related protein (PTHrP) produced by metastasizing cancer cells induces the expression of receptor activator of nuclear factor kappa-B ligand (RANKL) in bone tissue. RANKL in turn is a potent stimulator of osteoclasts and bone resorption. Osteoprotegerin (OPG) is a decoy receptor of RANKL which binds and eliminates RANKL.

Once a secondary tumor starts growing in the bone environment, its expansion is geometrically constrained by the presence of inelastic bone tissue. Physiologically, bone is remodeled through the process where old or damaged tissue is resorbed by cells specialized in bone destruction, osteoclasts, and new bone is produced by specialized bone-forming osteoblasts [3]–[5]. The RANK/RANKL/OPG pathway plays a crucial role in physiological bone remodeling. Receptor activator of nuclear factor kappa-B (RANK) is expressed by osteoclast precursors and mature osteoclasts. During remodeling, RANK ligand (RANKL) expressed by cells of the osteoblasic lineage stimulates osteoclast formation and directs osteoclasts towards sites of microdamage. Once osteoclasts have removed the old tissue, they move forward and recruit osteblasts, which in turn fill the previously resorbed trench with osteoid. The latter eventually mineralizes, and the process of mass-neutral bone renewal is complete. Mature osteoblasts also produce the soluble decoy receptor osteoprotegerin (OPG), which binds to RANKL and hence prevents it from interaction with RANK [3]. By producing OPG, osteoblasts have the ability to manipulate the RANKL concentration and gradient which control osteoclast allocation and steering [6]. Since cancer cells are unable to resorb bone, the only way for the tumor to expand is to trigger osteoclasts [7]. Cancer cells produce factors such as the parathyroid hormone-related protein (PTHrP), which induce the production of osteoclast-stimulating RANKL by osteoblasts, osteocytes and stromal cells [1]. The mostly membrane-bound RANKL binds to its receptor RANK, expressed on osteoclasts and their precursors, thus inducing osteoclast differentiation and stimulating resorptive activity. The resulting osteolysis provides in turn more space for the growing tumor – thereby closing the so-called ‘vicious cycle’ of bone resorption and tumor growth (Figure 1).

### Controversial results

Based on the model described above, the presence of osteoprotegerin in bone metastases should lead to reduced bone destruction and tumor growth. In agreement with this prediction, the systemic application of OPG leads to a decrease in tumor burden [8], and Corey and colleagues [9] demonstrated that OPG produced locally by cancer cells has a similar anti-metastatic effect. However, several lines of experimental evidence contradict the present model. First, it was repeatedly demonstrated that high circulating levels of osteoprotegerin in prostate cancer patients predict more bone metastases and more osteolysis [10], [11]. Even more interestingly, Fisher and colleagues [12] reported that local overexpression of osteoprotegerin by MCF-7 breast carcinoma cells co-expressing parathyroid hormone-related protein leads to increased osteolytic bone destruction and tumor growth in vivo - a result that appears to be in direct contradiction with the study of Corey and colleagues [9]. It has been suggested that the tumor-inducing effect of OPG is due to its inhibition of another ligand, TNF-related apoptosis-inducing ligand (TRAIL) [12]. TRAIL also acts as a modulator of osteoclast apoptosis [13] and differentiation [14]. However, it was shown that TRAIL cannot interfere with the anti-osteoclastogenic properties of osteoprotegerin [15], therefore OPG-TRAIL interactions cannot fully explain the enhanced osteolysis induced by OPG-overexpressing MCF-7 cells [12].

Altogether, these results indicate that osteoprotegerin plays a controversial role in bone metastases: while a large set of experimental data supports its osteoclast- and hence tumor-inhibiting role, in certain situations osteoprotegerin is documented to stimulate osteolysis and tumor growth.

### Hypothesis

We have recently demonstrated a potential role of OPG in enhancing RANKL gradients [6], which in turn are responsible for chemotactic movement of osteoclasts [16]. Based on these observations, we propose the following hypothesis regarding the action of osteoprotegerin in bone metastases: *1) When OPG is applied globally (i.e. systemically), it uniformly reduces RANKL levels, thus acting as an inhibitor of osteoclast formation and tumor growth. 2) When OPG is produced locally by cancer cells, the outcome is determined by the rate of OPG expression. At low expression rates, OPG enhances the chemotactic RANKL gradient responsible for osteoclast movement, thus stimulating osteolysis and tumor growth. At high expression rates, the RANKL-inhibiting effect of OPG becomes predominant and results in an overall decrease in osteolysis and tumor burden*. The distinction of the two regimes for tumor-derived osteoprotegerin provides a potential explanation of the differential experimental outcomes in [9] and [12].

To test this hypothesis we developed a mathematical model of tumor-osteoclast interactions, including the cytokine fields of RANKL, OPG and PTHrP, and examined the model predictions by means of appropriate *in silico* experiments focusing on the following main questions: 1) How does the impact of systemic OPG compare to the impact of cancer–cell derived OPG production? 2) How is indirect stimulation of RANKL production via PTHrP different from direct production of RANKL by tumor cells?

## Model

We adapted a previously developed nonlinear partial differential equations model of bone remodeling [6], [17] to include the spatio-temporal evolution of tumor mass, and the tumor-induced cytokine production. In this section, we summarize the modeling assumptions and introduce the model equations.

### Model assumptions

#### Geometry

**Figure 2:**
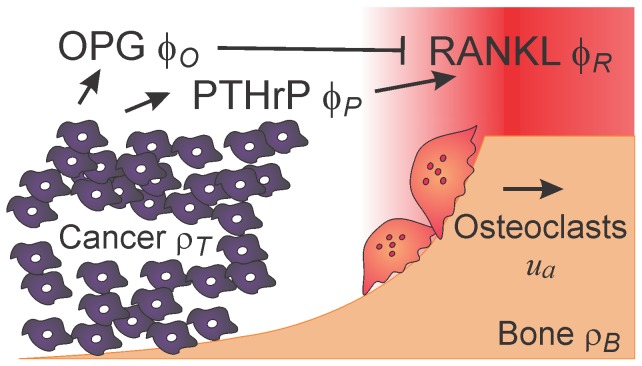
Model geometry. Active osteoclasts (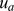) resorb bone (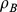) along the gradient (red) of the RANKL field (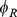), and move from left to right. The tumor (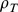) invades the space previously resorbed by active osteoclasts. Cancer cells produce PTHrP (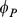), which diffuses and induces the expression of additional RANKL by osteoblastic bone cells. Cancer cells also produce OPG (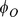) which diffuses, inhibits RANKL, and hence modifies the RANKL–gradient.

We model a single trabecula exposed to bone marrow and pre-existing cancer cells. Hemi-osteonal, trench-like remodeling of trabecular bone [4] reduces the geometry of the problem from three to two spatial dimensions, and assuming that the trabecula is locally flat, the model domain becomes a bounded subset of 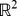. Assuming that the growing tumor induces a radially symmetric front of resorbing osteoclasts, we further reduce the model to one spatial dimension along the radial direction of movement (Figure 2). By choosing a unidirectional model of osteoclast propagation, we do not account for potential irregularities in the resorption path. In applied mathematics, it is a well-established methodology to study lower-dimensional versions of a model in question, especially because geometric complexity, potential blow-up phenomena and computational costs make the study of higher-dimensional versions much more involved. Even though the current one-dimensional framework is expected to be sufficient to test our hypothesis, it represents a significant simplication, and a two-dimensional evaluation of the model should be considered in the future.

#### Bone homeostasis

Metastasizing cancer cells cannot resorb bone tissue themselves and hence the outcome of the metastasis depends on the tumor's ability to stimulate osteoclasts. Even in the case of metastases which promote osteoblastic activity, bone resorption precedes bone formation [18]. The estimated rates of bone formation, 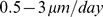 [4], [19], are smaller than the rates of bone resorption, 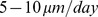 under physiological conditions [4]. In addition, in cancer patients, the bone resorption rates are estimated to further increase 4–10 fold [20], [21]. Since tumor growth is even faster than bone resorption (see paragraph on *cancer cells* below), we assume that the dynamics at the tumor–bone interface are dominated by the processes of bone resorption and tumor growth. Therefore, we focus here on the interaction of osteoclasts with the tumor, and do not take into account the much slower osteoblast dynamics.

We assume that osteoclastic differentiation and activity are controlled by the RANK/RANKL/OPG pathway. We assume that RANKL is produced by host tissue cells, including osteocytes, bone-lining cells, and stromal cells [22]–[25], and that it acts as a chemoattractant for osteoclast precursors [26] and active osteoclasts [16], [27]. RANKL stimulates osteoclast formation and activity, and prevents osteoclast death by acting through its receptor RANK, which is expressed on osteoclast precursors and mature osteoclasts [28]. Since trabecular bone is embedded in the bone marrow, we assume that there is a reservoir of osteoclast precursor cells, which then differentiate into active bone resorbing osteoclasts if sufficiently stimulated by RANKL. Finally, OPG is modeled as a soluble decoy receptor, whose sole action is to bind to RANKL. For a more detailed description of the assumptions underlying the model of bone homeostasis, we refer to *Text S1* as well as [6], [17].

#### Osteoclast initiation

The mechanisms underlying osteoclast initiation are complex and remain poorly understood to this date. In lieu of speculation, we assume that initiation has taken place prior to the beginning of the scenarios considered in this study, and that active osteoclasts are already present at time 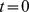.

#### Cancer cells

Tumor expansion is a complex process, and the mathematical modeling of tumor growth dynamics constitutes an active field of research [29]–[31]. However, growth rates of tumors outside the bone tissue have been shown to be of the order of 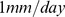 [32], which is 2–3 orders of magnitude faster than the rate of bone resorption (see previous paragraph on *bone homeostasis*). Therefore, the rate of tumor growth within the osseous tissue is expected to be limited by the availability of newly freed space, which in turn is limited by the rate of bone resorption. In keeping with this hierarchy of time scales, we assume that as soon as new space becomes available through bone resorption, proliferating cancer cells rapidly expand into the cavity. In other words, the tumor density 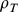 is described by a linear function of the bone density 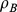: 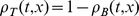. Note that both densities are rescaled to vary between 0 (no bone or tumor present) and 1 (space fully occupied by bone or tumor).

### Model equations

The mathematical model consists of 6 state variables: osteoclast population density (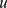), RANKL concentration (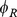), OPG concentration (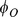), PTHrP concentration (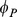), bone density (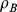) and tumor density (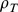). We introduce the model in several steps, and start with the osteoclast population density (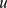). The dynamics of osteoclast formation and death are modeled as
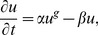
(1)where 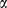 and 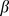 are formation and apotheosis rates, respectively, and the exponent 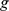 represents autocrine interactions among osteoclasts. We refer to [33], [34] for a complete discussion of such power-law models in the context of bone remodeling. Physiological parameters are such that equation (1) admits a stable fixed point 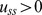, and we split the total osteoclast population 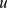 into 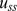 and a residual 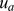, where 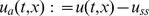. Note that 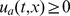 for all 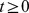, provided that 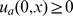 (see [17] for details). We regard cells below 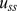 as inactive precursors, and consider an increase of 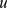 above 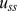 as differentiation of precursors into active, resorbing osteoclasts 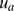. After adding the stimulation of osteoclast formation and chemotaxis by RANKL, we obtain the following evolution equation for the osteoclast population density:
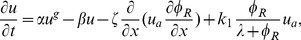
(2)where 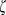 represents the chemotactic sensitivity of active osteoclasts to the RANKL gradient, 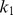 is the rate of osteoclast stimulation by RANKL, and the sigmoid function in the last term describes the half-saturation 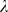 of the binding of RANKL to RANK receptors on osteoclasts. In general, 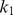 depends on the local bone density, as live osteoclasts have to attach to the bone surface [35]. We can usually relax this dependence due to the fact that the remodeling front is moving away from resorbed areas, and hence no active osteoclasts are present in areas that do not contain any bone tissue. Therefore, unless stated otherwise, 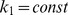. The bone density 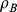, initially constant at 1, is degraded by resorbing osteoclasts (rate 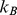) as 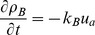. As explained in *Model Assumptions*, the tumor density 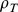 is described as 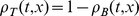. The dynamics of the RANKL field 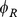 are governed by production by cancer cells (rate 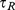), diffusion (rate 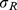), degradation (rate 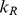) and binding to RANK receptors on active osteoclasts (rate 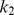, half-saturation 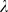),
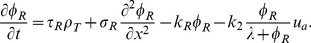
(3)We assume that the concentration of membrane-bound RANKL is kept constant on expressing cells, and hence we neglect its decay rate, i.e. we set 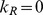. To reconcile the known osteolysis-inhibiting effects of systemically administered osteoprotegerin [8], and osteolysis-inducing effects of OPG locally produced by metastasizing cancer cells [12], we extend the model to account for OPG produced locally by cancer cells. The evolution equation for the OPG concentration 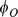 includes expression by cancer cells (rate 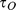) and systemic sources (rate 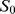), diffusion (rate 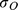), degradation (rate 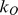) as well as binding to RANKL (rate 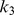),
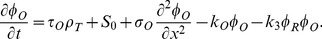
(4)The last term on the right-hand side of (4) is also added to the RANKL equation (3). It is well-established that cancer cells metastasizing to bone commonly produce a mediator, such as parathyroid hormone-related protein, which in turn promotes RANKL production by osteoblastic and stromal cells [1], [36], [37]. To model this scenario, we introduce the PTHrP concentration 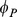 as a new state-variable: once produced by cancer cells (rate 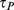), PTHrP diffuses across the tissue (rate 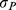) and is degraded by proteases (rate 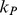),
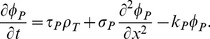
(5)While diffusing across the tissue, PTHrP induces the expression of RANKL by osteoblastic cells in the bone tissue, and we describe this by adding a source term (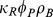) to the RANKL equation (3). Finally, combining equations (2), (3), (4) and (5) with the modifications and extensions described in the text, the complete system of equations reads
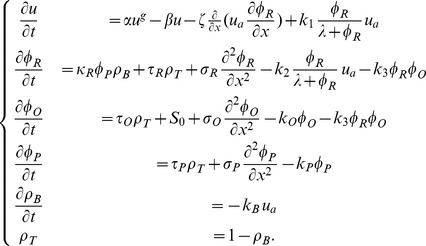
(6)The variables and model parameters are summarized in Table 1, and a visualization of the spatial distribution of the fields is found in Figure 2.

**Table 1:** Variables and parameters in model (6).

Variable	Description
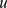	Density of osteoclasts
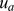	Density of active osteoclasts
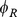	RANKL concentration
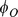	OPG concentration
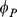	PTHrP concentration
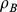	Bone density
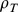	Tumor density

### Initial conditions, boundary conditions and numerical analysis

**Figure 3:**
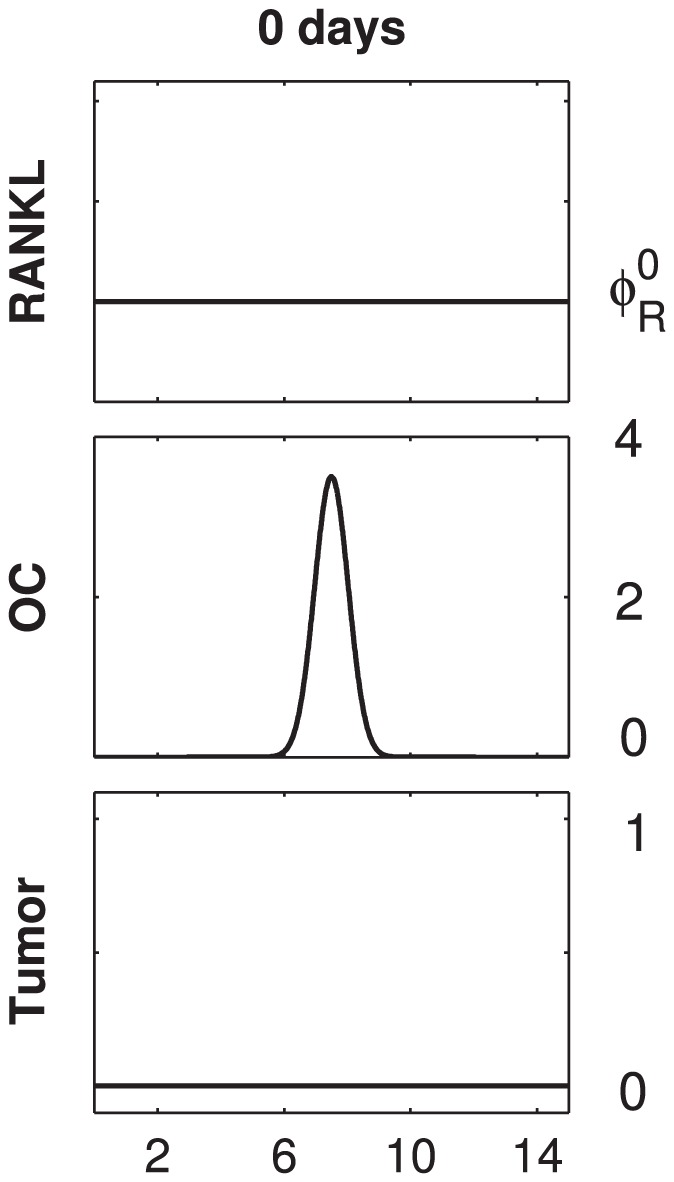
Initial conditions. The set of initial conditions used for all simulations of the study. The initial RANKL field consists of host–tissue RANKL only, and is of constant concentration 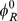. The initial profile of active osteoclasts (OC) is placed in the middle of the domain. Initially, there is no tumor present. Not shown above are the following fields: the bone tissue is intact, i.e. 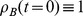, and the OPG and PTHrP concentrations are uniformly zero. Note that the initial conditions are consistent with the choice of periodic boundary conditions.

The initial RANKL field consists of host-tissue RANKL only, and is of constant concentration 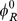. The initial profile of active osteoclasts is placed in the middle of the domain, and there is no tumor present (see Figure 3). The initial bone tissue is intact, 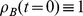, and the OPG and PTHrP concentrations are uniformly zero.

**Figure 4:**
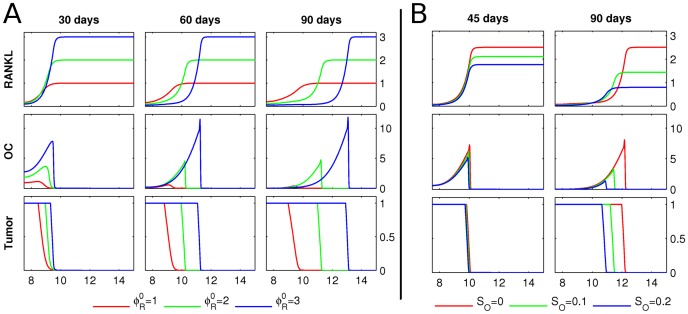
Host tissue RANKL and systemic OPG. **A** Starting from the initial conditions described in Figure 3, the RANKL concentration, osteoclast population density (OC) and tumor density (Tumor) are shown at 30, 60 and 90 days, respectively. The outcomes for three different values of the host-RANKL level 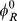 are shown. The computational domain is 15 mm long, but since the fields are symmetric, only the right half is shown. The y-axes have the following units: RANKL in pmol/mm; OC in cells/mm; tumor density is normalized between 0, when there is no tumor per unit length, and 1, when the unit space is fully occupied by tumor. The resorption fronts of osteoclasts either reach wave-like propagation (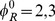) or die out (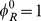). **B** Starting from the initial conditions described in Figure 3, the evolution of the RANKL concentration, osteoclast population density (OC) and tumor density (Tumor) is shown after 45 and 90 days, respectively. The initial host-RANKL level is 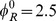, and between 20 and 90 days, a uniform source of OPG is administered at 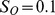 (green) and 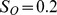 (blue), respectively. Compared to the control at 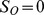 (red), the respective tumor burdens are reduced.

**Figure 5:**
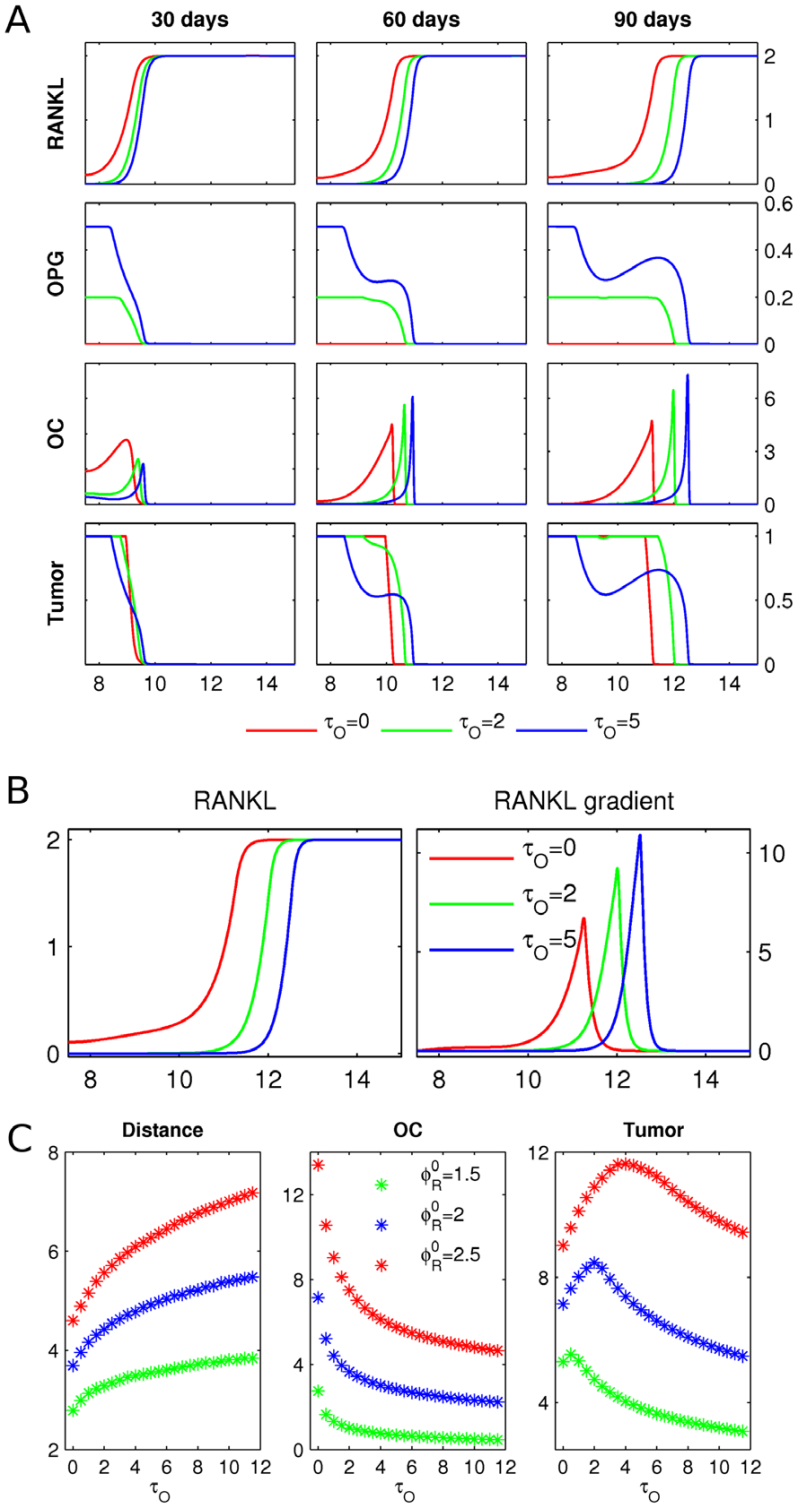
OPG production by tumor. **A** Starting from the initial conditions described in Figure 3, the RANKL and OPG concentrations, the osteoclast population density (OC) and the tumor density (Tumor) are shown after 30, 60 and 90 days, respectively. The growing tumor produces OPG at rates 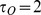 (green) and 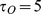 (blue), with a control case 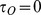 (red). Length of domain is 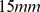, and only the right halves of the symmetric fields are shown. Scales are as in Figure 4, and OPG is in pmol/mm. **B** *Left:* zoom in on RANKL at 90 days in panel A. *Right:* the RANKL gradients are obtained by taking the spatial derivatives of the respective fields. **C** The simulation described in panel A is repeated for different initial RANKL levels 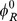, and different levels of OPG production by cancer cells 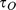. After 90 days, the following quantities are shown: distance traveled by osteoclasts (Distance), total number of active osteoclasts (OC), and total tumor mass (Tumor).

**Figure 6:**
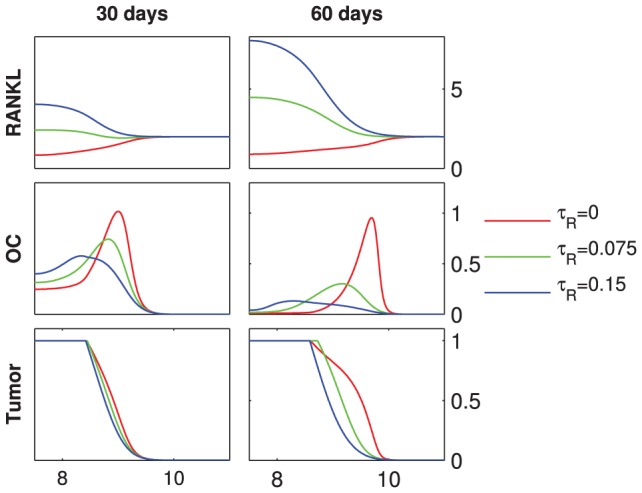
Direct RANKL production by tumor. Starting from the initial conditions described in Figure 3, the RANKL concentration, the osteoclast population density (OC) and the tumor density (Tumor) are shown at 30 and 60 days, respectively. The initial host-tissue level of RANKL is 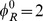. For 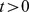, RANKL is produced by the tumor at varying rates 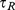. Length of domain is 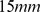, only the right halves of the symmetric fields are shown, the units of the y-axes are as in Figure 4.

**Figure 7:**
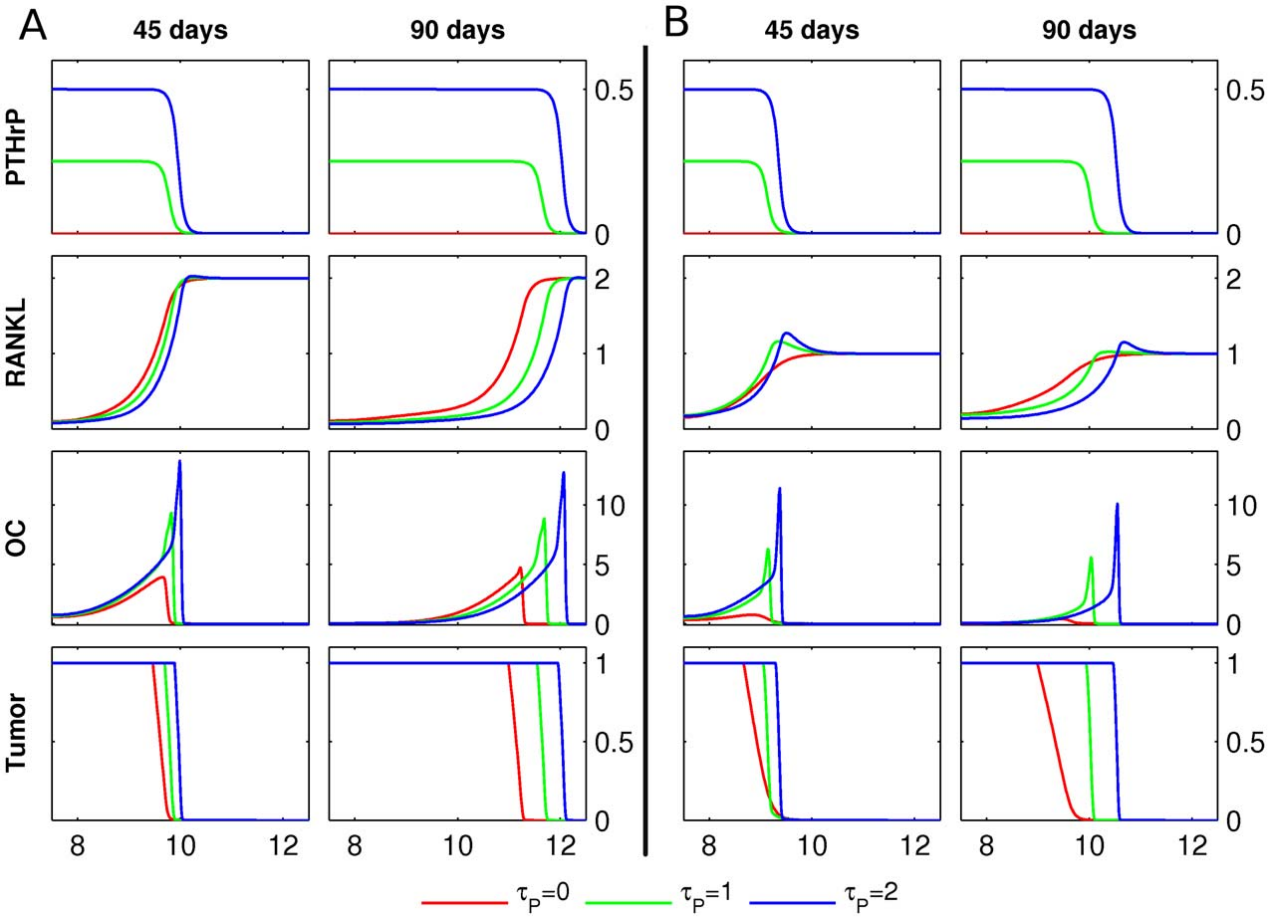
PTHrP production by tumor. **A** Starting from the initial conditions described in Figure 3, the PTHrP and RANKL concentrations, the osteoclast population density (OC) and the tumor density (Tumor) are shown at 45 and 90 days, respectively. The initial host–tissue level of RANKL is 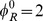. For 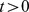, tumor produces PTHrP at rates 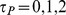. Length of domain is 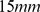, only the right halves of the symmetric fields are shown, the units of the y-axes are as in Figure 4, and PTHrP has units 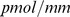. **B** The simulations in A were repeated for the initial host tissue level of RANKL 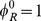.

**Figure 8:**
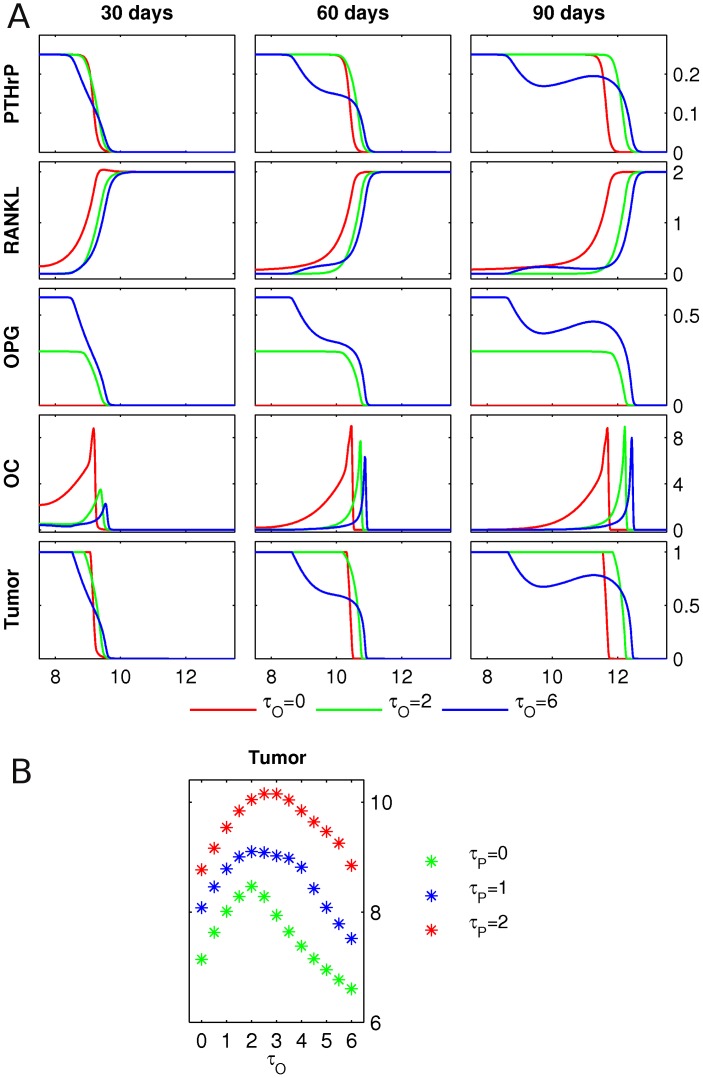
PTHrP and OPG production by tumor. **A** Starting from the initial conditions described in Figure 3, the PTHrP, RANKL and OPG concentrations, the osteoclast population density (OC) and the tumor density (Tumor) are shown at 30, 60 and 90 days, respectively. The initial RANKL level is 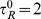. The growing tumor produces PTHrP at a fixed rate 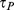, and three different levels of tumor-derived OPG production 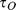 are considered. Length of the domain is 15 mm, only the right halves of the symmetric fields are shown. Units of the y-axes are as in Figure 7, and the OPG field has units of 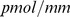. **B** The simulation described in panel A is performed for varying values of 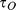 and 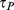, and the total tumor mass at 90 days is presented.

The results presented in Figures 4–8 are based on numerical solutions of different versions of system (6), together with periodic boundary conditions and initial conditions as specified above. The parameter values are matched to in vivo observations where available, and a tuning method is applied to the set of unmatched parameters as explained in *Text S1*. The time stepping is performed with a fractional step method as described in [38]. Thereby, adaptive Runge-Kutta solvers are used for the advection and reaction parts, and a TR-BDF2 solver for the diffusion parts. Spatial discretisations are performed by means of finite differences (chemotactic term) and spectral collocation (diffusion terms). See *Text S1* for details.

## Results

We performed five different numerical experiments, referred to as *scenarios*, in order of increasing complexity. In *Scenario 1*, the impact of host-tissue RANKL and systemic OPG application on tumor growth are analyzed. Then, the case where cancer cells express OPG locally is the subject of *Scenario 2*. The impact of cancer-derived RANKL is studied in *Scenario 3*, and PTHrP expression by cancer cells (in absence of local OPG production) is the subject of *Scenario 4*. Finally, the most comprehensive situation is captured in *Scenario 5*, where cancer cells produce both PTHrP and OPG.

### Scenario 1: Host tissue RANKL and systemic OPG

We first assess how different levels of RANKL in the host tissue influence tumor growth. We solve equation (6) in absence of the OPG (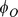) and PTHrP (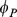) fields, and we set 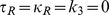 in the 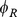-equation. The host-tissue level of RANKL, 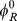, is modeled in the initial RANKL field, i.e. we set 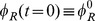, see Figure 3. It is important to note that the parameter 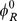 denotes the concentration of active RANKL, i.e. the total concentration of tissue-derived RANKL minus the concentration of RANKL which is bound to OPG. In agreement with the known action of RANKL as a potent stimulator of osteoclast differentiation [28], we observe a positive correlation between RANKL levels and tumor growth (Figure 4-A). At first, the initial osteoclast profile splits up symmetrically into two individual resorption fronts (note that since the field is symmetric, we only depict the right half of the modeling domain). The resorption front propagates in a wave-like manner in the case of sufficiently high RANKL levels (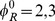), or dies out in the case of insufficient stimulation by RANKL (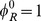). This suggests the existence of a threshold concentration of RANKL necessary for a sustainable resorption event.

Next, we investigate the impact of systemically administered OPG by introducing equation (4) with 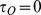 and a spatially uniform systemic source 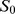. We assume that the OPG administration only starts after 20 days of tumor growth, and that the source is then continuously applied until the end of the simulation. The resulting evolution of the fields is depicted in Figure 4-B. As expected, systemic application of OPG considerably decreases the tumor burden after 90 days.

The simulations in Figure 4 are relevant for two aspects of osteolytic bone metastases. 1) The tissue RANKL level is known to positively correlate with bone metastases [39], and tumors preferentially metastasize to actively remodeled skeletal sites, likely containing higher RANKL levels [40], [41]. 2) Systemic application of osteoprotegerin, which binds to RANKL in the bone tissue, lowering its levels, is known to inhibit osteolysis associated with cancer metastases to bone [8].

### Scenario 2: OPG production by tumor

Next, we assess how the local production of OPG by cancer cells affects the progression of bone metastases. We consider system (6) in absence of the PTHrP equation, set 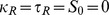, and repeat the same scenario for varying levels of osteoprotegerin production 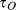, see Figure 5-A (a dynamic representation of these simulations is found in *Video S1*). In comparison to the control case with no OPG expression (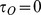), higher levels of OPG production by cancer cells (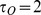 and 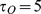) lead to an increase in osteoclast advance (see OC after 90 days), and hence a bigger resorption area. A closer look at the RANKL field after 90 days in Figure 5-B reveals that tumor-produced OPG removes residual RANKL left behind the remodeling front, resulting in the formation of steeper RANKL gradients, and hence increased speed of osteoclast migration. Note that the RANKL gradients of 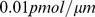 in our simulations are consistent with the gradients of 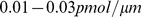 which were shown to induce osteoclast chemotaxis in experimental studies [16]. In Figure 5-C, we present a systematic study of the effect of OPG production by cancer cells on osteoclast migration, the number of active osteoclasts and tumor mass. These results demonstrate that the interplay of two main factors is important in determining the overall outcome of OPG action. First, the OPG-induced increase in RANKL gradient and osteoclast speed (evident by the distance traveled in 90 days) is accompanied by a decrease in the number of active osteoclasts. This results in a non-trivial dependence of the tumor mass on the rate of OPG production by cancer cells. While low and intermediate expression of osteoprotegerin by cancer cells correlates with an increase in osteolysis and hence tumor burden, at high OPG expression, the remodeling front is too small to completely resorb all bone tissue, leading to an overall decrease in tumor mass. Second, the effect of tumor-produced OPG strongly depends on the levels of RANKL in the bone tissue: at low RANKL levels, OPG is predominantly inhibitory, while at high RANKL levels, tumor-produced OPG becomes more effective in inducing osteolysis (Figure 5-C, compare 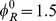 and 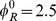). Thus, the model predicts the existence of two different regimes for the impact of tumor-produced OPG, which correspond well to experimental findings of inhibition of osteolysis by cancer cell–produced OPG [9], and stimulation of osteolysis by cancer cell–produced OPG [12].

### Scenario 3: Direct RANKL production by tumor

Since high levels of RANKL in the tissue are important for the osteolysis-enhancing effects of OPG, we assess now if cancer cells could promote osteolysis by directly producing RANKL. We model this situation by adding a tumor-derived RANKL source to the 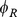-equation, i.e. we solve system (6) in absence of the OPG and PTHrP fields, set 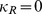, and repeat the same scenario for varying values of 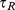. Note in particular that for this scenario it is necessary to model the osteoclast-stimulation rate 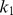 to be dependent on the bone density (see *Text S1* for details), i.e. we replace the reaction term in the osteoclast equation of (6) by
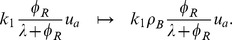
As shown in Figure 6, the tumor-derived production of RANKL leads to a reversal of the RANKL gradient. Rather than moving away from the tumor and resorbing more bone to provide new space for proliferating cancer cells, osteoclasts move towards the tumor. Consequently, no traveling remodeling front is formed, osteolysis is disrupted, and tumor growth decreases with increase in RANKL production rate 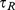. Although the RANK-RANKL dynamics are known to play an important role in bone metastases [16], [39], there is uncertainty regarding the actual source of RANKL. While some studies report direct expression of RANKL by metastasizing squamuous cell carcinoma and prostate cancer cells [42], [43], others suggest that there is no direct production of RANKL by cancer cells [44], [45]. In addition, it has been shown that breast cancer cells cease to express RANKL upon embedding into the bone environment [46]. Our simulations suggest that expression of RANKL does not provide cancer cells with an advantage in the bone microenvironment.

### Scenario 4: Indirect RANKL production via PTHrP

We consider now the case where cancer cells produce PTHrP (but no OPG), which in turn promotes RANKL production by osteoblastic and stromal cells. More precisely, we consider system (6) in absence of the 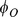-equation, set 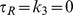, and repeat the same scenario for varying values of PTHrP production 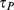. If the initial tissue level of RANKL is sufficient for the formation of a traveling wave-like front of osteoclasts in the absence of PTHrP production (Figure 7-A, 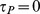), switching on the PTHrP production leads to faster and bigger resorption fronts, and hence a further increase in tumor mass after 90 days (Figure 7-A, 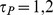). Moreover, if the initial RANKL level is insufficient to sustain a traveling resorption front (Figure 7-B, 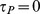), the expression of PTHrP by cancer cells induces a traveling wave of active osteoclasts, and hence an increase in tumor mass (Figure 7-B, 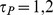). Thus, in good agreement with experimental data [1], [12], the tumor is able to efficiently promote its own growth by producing PTHrP.

### Scenario 5: OPG and PTHrP production by tumor

We assess now the impact of simultaneous production of OPG and PTHrP by cancer cells. This leads to the most comprehensive scenario considered, and is captured by the complete system (6) with 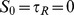. First, we study osteolysis and tumor growth for varying tumor-derived osteoprotegerin production rates (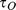) at a fixed level of PTHrP production (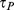), see Figure 8–A. An increase in osteoprotegerin production 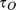 leads to an enhanced RANKL gradient, and the resulting increase in the speed of the remodeling front is accompanied by an increase of the resorbed area and a decrease in the number of active osteoclasts. A systematic study of the impact of varying OPG and PTHrP production rates on the tumor mass, see Figure 8-B, reveals that at low to intermediate OPG expression rates by cancer cells (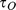), there is an increase in overall tumor burden after 90 days, for all levels of PTHrP production (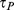). On the other hand, high levels of OPG expression lead to a decrease in tumor burden, which eventually drops below the value for 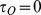.

### Meta-Analysis of OPG, PTHrP and RANKL gene expression

**Figure 9:**
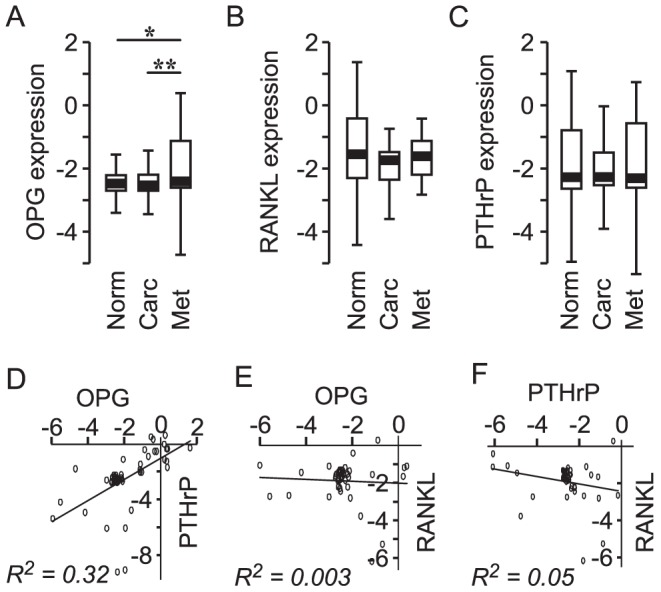
OPG, RANKL and PTHrP expression in prostate cancer. Data from nine gene expression data sets [47]–[55] were combined and analyzed. **A–C** Expression of OPG (A), RANKL (B) and PTHrP (C) are shown in the box-plots where the lower whisker indicates the 1st percentile, the limits of the box indicate the 25th and 75th percentiles, and the upper whisker indicates the 99th percentile. Statistical significance is indicated by 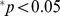, 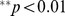, calculated using one-way ANOVA. **D–F** Data for the metastatic prostate samples were analyzed for the correlation in the expression of OPG and PTHrP (D), OPG and RANKL (E), and RANKL and PTHrP (F).

We examined the expression of OPG, RANKL, and PTHrP in patient samples from normal prostate tissue, prostate carcinoma, and metastatic prostate carcinoma tissues, as reported in the studies [47]–[55]. We used the publicly available gene expression data analysis engine Oncomine Research Edition (www.oncomine.org), and processed data as described in *Text S2* for details. We found that expression of osteoprotegerin was significantly increased in samples from metastatic prostate cancer compared to normal prostate (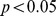), as well as prostate carcinoma (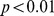) (Figure 9-A). In contrast, expression of RANKL and PTHrP did not exhibit significant changes (Figures 9-B and 9-C).

Since our simulations suggest that most effective in promoting bone metastases is the combination of OPG and PTHrP, we further assessed the correlation between the expression of OPG, PTHrP and RANKL in samples from metastatic prostate carcinoma only. We found that expression of osteoprotegerin by metastatic prostate cancer cells exhibited significant positive correlation with PTHrP, 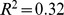 (Figure 9-D), while no correlation was found between OPG and RANKL, 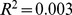 (Figure 9-E) or PTHrP and RANKL, 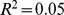 (Figure 9-F). Thus, consistent with our modeling findings, gene expression data demonstrate an increase in OPG, rather than RANKL, in metastatic prostate cancer, as well as a positive correlation between the expression of OPG and PTHrP.

## Discussion

The goal of this study was to propose and test a novel hypothesis explaining the differential and seemingly contradictory experimental results regarding the role of osteoprotegerin in bone metastases. Whereas systemic application of osteoprotegerin is known to decrease osteolysis and tumor growth [8], two similar experiments have shown that osteoprotegerin produced locally by metastatic cancer cells in the bone environment can lead to a decrease [9] or an increase [12] in osteolysis and tumor growth. Given the well-established role of osteoprotegerin as an osteoclast inhibitor [56], the outcome of systemic osteoprotegerin application does not bear any surprises, but the osteolysis promoting effects in [12], as well as the increased osteolysis in metastatic cancer patients with high levels of circulating osteoprotegerin [10], [11], appear to be contradictory. To resolve this apparent contradiction, we proposed that the spatial configuration of the tumor-bone interface in conjunction with the magnitude of tumor-derived osteoprotegerin expression determines the resulting effect of OPG. We hypothesized the existence of two distinct dynamical regimes for locally produced osteoprotegerin: (1) at low expression rates, tumor-produced OPG primarily enhances the chemotactic RANKL gradient oriented towards the unresorbed bone tissue, thus stimulating osteoclast movement, and resulting in an increase in osteolysis and hence tumor mass. (2) at high expression rates of tumor-derived OPG, the RANKL-inhibiting effect of OPG becomes predominant and results in an overall decrease in tumor burden. Based on a previously presented mathematical model of bone remodeling [6], [17], we developed a nonlinear partial differential equations model describing the interactions between metastatic cancer cells and the bone environment. In good agreement with our hypothesis, the model suggests the existence of two distinct dynamic regimes where tumor growth is either accelerated or slowed down by cancer-derived osteoprotegerin. These observations are further substantiated by a meta-analysis of gene expression, which shows that osteoprotegerin expression in metastatic prostate tissue is increased compared to normal prostate and prostate carcinoma samples.

The model simulations point out another interesting aspect related to the spatial configuration of the tumor-bone interface. Our model predicts that the direct expression of osteoclastogenic cytokine RANKL by cancer cells may result in a reversal of the chemotactic gradient, thus slowing down osteolysis and tumor growth. The model suggests that it is crucial for cancer cells to express a mediator (such as parathyroid hormone-related protein) that diffuses across the tissue before triggering the expression of RANKL on osteoblastic cells. The involvement of such a mediator assures that the RANKL gradient is correctly oriented to induce osteoclast movement away from the tumor into unresorbed bone tissue. In accordance, the meta-analysis of gene expression reveals that osteoprotegerin expression in metastatic prostate tissue is positively correlated with the expression of PTHrP, but not RANKL.

Due to the large number of a priori unknown parameters in the model, our results are predominantly of qualitative nature. While the emergence of two distinct regimes of OPG action is observed across a large span of parameter values, and is in particular independent of the production rate of parathyroid hormone-related protein PTHrP by cancer cells, further experimental investigation will be indispensable for a full validation of our hypothesis. Thereby, an experimental assessment of the diffusion rates of different molecules in tumor and bone tissues would be useful. In addition, the potential role of OPG in stimulating osteoclast movement could be studied in vitro, and the metastatic capacity of prostate cancer cells expressing different levels of OPG could be assessed in vivo. In the meantime, our qualitative predictions are valuable in suggesting a new conceptual model which is consistent with all the experimental data available to date. In particular, our model suggests that future experimental designs should take into account the directional movement of the constituent cells, as well as the geometry of the tumor-bone interface.

The proposed mechanism emphasizes the importance of the spatial configuration of molecular densities, and thus may be relevant to other systems where distinct spatial patterns are imperative. An interesting example is the regulation of immune cell migration by chemokines. It has recently been shown that in addition to signaling receptors, there exist several decoy receptors that bind to chemokines, but do not induce any cellular changes [57]. Our hypothesis suggests that a potential role for these decoy receptors is the creation and enhancement of chemokine gradients. Another example is the difference in action of tumor-produced and host tissue-produced angiogenic factors, such as nitric oxide [58], which is in agreement with the importance of spatial coordination of tumor vascularization for tumor growth at the metastatic site. In summary, our study demonstrates that taking into account the spatial distribution of regulators, receptors and decoy receptors can reveal novel mechanisms inaccessible to conventional models based on global regulator-receptor ratios.

## Supporting Information

Text S1 Model development, numerics, and parameter estimation.
(PDF)

Text S2 Meta-analysis of gene expression.
(PDF)

Video S1 Dynamics of Scenario 2 as described in the caption of Figure 5A.
(AVI)
