# Supplementary material for: Osteoprotegerin in Bone Metastases: Mathematical Solution to the Puzzle
Source: PLoS Comput Biol. 2012 Oct 18;8(10):e1002703. doi: 10.1371/journal.pcbi.1002703 (PMC3475686; doi:10.1371/journal.pcbi.1002703)
Supplement: Text S1 — Model development, numerics, and parameter estimation. (PDF) [file pcbi.1002703.s001.pdf]

Supporting Information I

OPG in bone metastases: mathematical solution to the puzzle

Marc D. Ryser[1,†], Yiding Qu[2], Svetlana V. Komarova[3,*]

1 Department of Mathematics and Statistics, McGill University, Montréal, Québec, Canada
2 Department of Anatomy and Cell Biology, McGill University, Montréal, Québec, Canada
3 Faculty of Dentistry, and Department of Anatomy and Cell Biology, McGill University, Montréal, Québec, Canada

∗ **E-mail: svetlana.komarova@mcgill.ca**
† **Current address: Department of Mathematics, Duke University, Durham, NC, USA**

1 Model development

1.1 Previous Work

The modeling part of this work is based on a previously developed model of bone homeostasis introduced in [1,2]. This model describes the spatio-temporal evolution of osteoclasts and osteoblasts as well as their interactions through the RANK/RANKL/OPG pathway, and can be summarized as follows

$$\begin{cases} \partial_t u_1 &= \alpha_1 u_1^{g_{11}} - \beta_1 u_1 - \zeta \nabla \cdot (y_1 \nabla \phi_R) + k_1 \ \frac{\phi_R}{\lambda + \phi_R} \ \theta(y_1) \ u_1 \\ \partial_t u_2 &= \alpha_2 u_1^{g_{12}} - \beta_2 u_2 \\ \partial_t \phi_R &= a_R \ y_{2,t_R} + \kappa_R \Delta(\phi_R^{\epsilon_R}) - k_2 \ \frac{\phi_R}{\lambda + \phi_R} \ \theta(y_1) \ u_1 - k_3 \phi_R \phi_O \\ \partial_t \phi_O &= a_O \ y_{2,t_O} + \kappa_O \Delta(\phi_O^{\epsilon_O}) - k_3 \phi_R \phi_O \\ \partial_t z &= -f_1 \ y_1 + f_2 \ y_2. \end{cases} \tag{1}$$

Featured in (1) are the following state variables: osteoclasts (u_1), osteoblasts (u_2), the RANKL field (ϕ_R), the OPG field (ϕ_O) and the bone mass (z), which is ρ_B in the current model. The fields y_i are the active cell populations and differ from the total cell populations u_i by an additive constant (see discussion in main text). Note that θ is the Heaviside function ($\theta(x) = 1$ for $x > 0$ and $\theta(x) = 0$ otherwise), and $y_{2,\tau}$ is a delay term defined by

$$y_{2,\tau} = e^{-\beta_2 \tau} y_2(t - \tau), \qquad \tau > 0. \tag{2}$$

Among the various pathways involved in the remodelling process, only the RANK/RANKL/OPG pathway is modeled explicitly, the other pathways are captured by the nonlinearities in the u_i equations. We discuss now the improvements and modifications of (1) that lead to the models used in *Scenarios 1-5* in this study.

1.2 Osteoclast stimulation by RANKL

RANKL activates and stimulates osteoclasts by binding to RANK receptors on their surface. In (1), this ligand-receptor interaction is captured in the last term of the osteoclast equation as well as the third term in the RANKL equation,

$$\pm k_i \frac{\phi_R}{\lambda + \phi_R} \theta(y_1) \ u_1. \tag{3}$$

Note that we have two different reaction rates k_1, k_2 to account for the reversibility of the RANK-RANKL binding: since a ligand can contribute to the stimulation of a cell without getting permanently bound to the receptor, we generally have $k_2 < k_1$. The dependence on $\theta(y_1)u_1$ in (3) ensures that only osteoclasts in the vicinity of the remodeling front of active cells are stimulated by RANK-RANKL interactions. Even though this choice lead to satisfying results in previous studies, we chose to replace it as $\theta(y_1)u_1 \mapsto y_1$. The reason for this choice is twofold: first, the dependence on $\theta(y_1)u_1$ implied that precursor cells responded to RANKL when close to the remodeling front, but not when further away from it. In absence of experimental evidence for such a functional distinction, the choice y_1 seems more meaningful. Second, from a mathematical point of view the use of the Heaviside function is expected to decrease the regularity of the solutions and hence by replacing it with a linear dependence as $\sim y_1$, the regularity of the solution is increased, and in particular the problem becomes more tractable from a numerical perspective.

Another issue with the RANK-RANKL term (3) is the proportionality constant k_1: in fact, active osteoclasts are at all times attached to the bone surface and this implies that the activation of osteoclastic activity should vanish as the local bone density ρ_B goes to zero. Despite these observations, the choice of keeping k_1 constant throughout this study (except for *Scenario 3*) can be justified *a posteriori* by observing that the RANKL gradients are consistently guiding the active osteoclasts away from previously resorbed spots (see e.g. Figure 8-A in main text). In *Scenario 3* however, the RANKL gradient is reversed and guides osteoclasts towards the already resorbed centre of the domain (see Figure 6 in main text). Therefore, we modeled the linear dependence $k_1 \sim \rho_B$ explicitly to avoid physiologically unfeasible situations as discussed in [2].

1.3 Chemotaxis

Chemotactic movement of osteoclasts has been documented in several studies, see e.g. [3], as well as [4] and [5]. Since *distressed* osteocytes have been shown to express RANKL [6,7], it seems plausible that there is a RANKL gradient away from damaged sites (e.g. microfractures). Interestingly though, there need not be a predefined RANKL gradient in the tissue: as illustrated by Figure 3-A in the main text, which starts with a homogenous RANKL field, the binding of RANKL to RANK receptors is sufficient to create a local gradient, which in turn drives the osteoclast movement. Furthermore, as pointed out in Scenario 4, there need not even be a sufficient RANKL background level at time $t = 0$: the production of PTHrP by the tumor is sufficient to induce the necessary RANKL for osteoclast stimulation and chemotaxis. In conclusion, the outcome of the simulations is largely independent of a pre–existing RANKL gradient in the tissue, and relies only on the well–documented properties of osteoclastic stimulation and attraction by RANKL.

1.4 Porous Diffusion

In the homeostasis model (1) we assumed ϕ_R to obey porous diffusion dynamics, i.e. we allowed for exponents $\epsilon_R > 1$. The main difference between regular diffusion ($\epsilon = 1$) and porous diffusion ($\epsilon > 1$) becomes apparent when considering the model equation on $\mathbb{R}$,

$$\frac{\partial \phi(t,x)}{\partial t} = \frac{\partial^2 \phi^\epsilon(t,x)}{\partial x^2}. \tag{4}$$

The dynamics of (4) with $\epsilon = 1$ have infinite propagation speed, i.e. for any $t > 0$ the solution ϕ will have infinite support, even if the initial field was compactly supported. On the other hand, solving (4) with $\epsilon > 1$, compactly supported initial conditions will remain so for all $t > 0$, see [8]. Even though the choice of porous diffusion seems more meaningful for a mainly membrane bound cytokine such as RANKL, when we repeated all the simulations in this study for $\epsilon_R = 1.5, 2, 2.5$, we observed no qualitative differences

to the case $\epsilon = 1$. This observation, combined with the numerical advantage of working with a linear diffusion term, lead us to set $\epsilon_R = 1$ in this study.

1.5 Finite Osteoclast Speed

As observed in Figure 8A, tumour-derived OPG leads to an increase in osteoclast migration speed: the distance traveled by the remodeling front during 90 days monotonically increases with increasing OPG production τ_O. The present model does not account for an upper limit as to how fast osteoclasts can move across the trabecula. Osteoclasts have been shown to travel at a speed of $\sim 10\mu m$ per day in physiological settings, and 5–10 times faster in cancer patients [9, 10]. Since the maximal distance travelled by the cutting cone in our simulations is 7 mm in 90 days (Figure 5C in main text), the numerical experiments reported in this study are within the range of realistic osteoclast speeds.

1.6 Undirected Osteoclast Motility

Many chemokines are known to induce random cell motility in addition to directional cell migration. To our knowledge, there is no experimental evidence that RANK-RANKL binding leads to an increase in undirected motility of osteoclasts. Nevertheless, we wanted to make sure that the nonlinear relationship

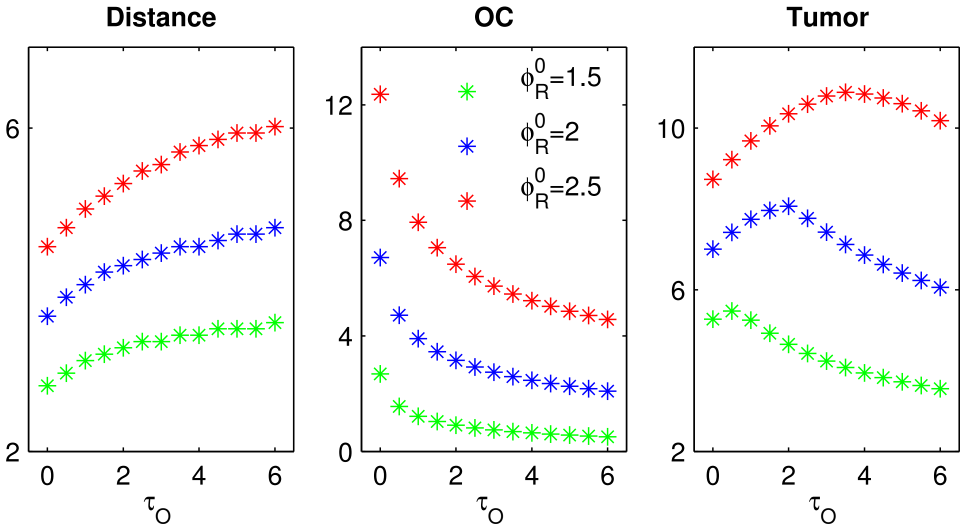

Figure 1. Undirected osteoclast motility. Potential stimulation of random osteoclast motility by RANKL does not result in qualitative differences in model outcome. The set of simulations in Figure 5C of the main text are repeated, this time accounting for undirected motility of osteoclasts. The simulation is repeated for different values of initial RANKL ϕ_R^0, and different levels of OPG production by cancer cells, τ_O. After 90 days, the following quantities are shown: distance traveled by osteoclasts (Distance), total number of active osteoclasts (OC), and total tumor mass (Tumor).

of tumor burden and OPG production in Figures 5C and 8B of the main text would also be observed if diffusive movement of osteoclasts did occur. In order to account for diffusive movement of osteoclasts, we replaced the osteoclast equation in system (6) of the main text by

$$\frac{\partial u}{\partial t} = \alpha u^g - \beta u - \zeta \frac{\partial}{\partial x}(u_a \frac{\partial \phi_R}{\partial x}) + k_1 \frac{\phi_R}{\lambda + \phi_R} u_a + \sigma_u \frac{\partial u_a^2}{\partial x^2}. \tag{5}$$

The new parameter σ_u represents the diffusive motility of osteoclasts. In absence of experimental estimates, we used the tuning strategy for free parameters (as described in Section 3 below) to determine σ_u. We set $\sigma_u = 10^{-3} mm^2 d^{-1}$ since we found that higher values of σ_u lead to osteoclast populations that moved too fast and did not stay compactly supported. We next assessed how osteoclast movement and tumor growth were affected by the changes in OPG production by tumor cells, and compared predictions of the model with osteoclast motility to the model without osteoclast motility (presented in Figure 5 in the main text). We found that accounting for osteoclast diffusion leads to a qualitatively similar behavior: the tumor burden remains a bell-shaped function of the OPG production rate τ_O (Figure 1 of SI Text 1). In view of these observations, we decided to avoid unnecessary complexity and uncertainty in our model, and set $\sigma_u = 0$ for all simulations in the main text.

1.7 Reversibility of RANKL–OPG binding

We assumed that the binding of RANKL and OPG is irreversible, since to our knowledge, there is no experimental evidence that suggests otherwise. Nevertheless, to make sure that the possible existence of

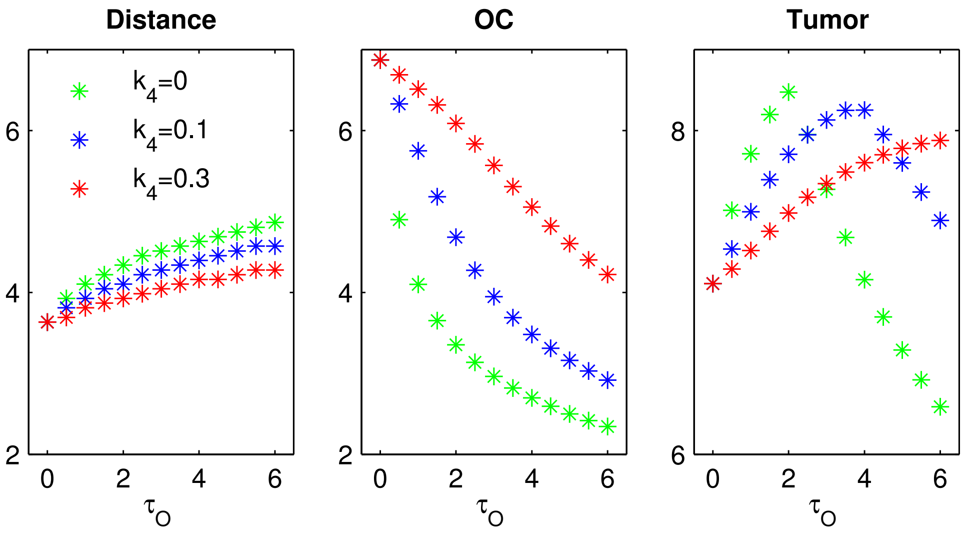

Figure 2. Reversibility of RANKL/OPG binding. Potential reversibility of RANKL/OPG binding does not result in qualitative differences in model outcome. The set of simulations in Figure 5C of the main text are repeated, while accounting for reversibility of the formation of [RANKL/OPG] complexes. The tissue level of RANKL is $\phi_R^0 = 2$, and the simulation is repeated for different values of k_4 (RANKL-OPG dissociation rate), and different levels of OPG production by cancer cells, τ_O. After 90 days, the following quantities are shown: distance traveled by osteoclasts (Distance), total number of active osteoclasts (OC), and total tumor mass (Tumor).

reversibility would not lead to qualitatively different results, we re-examined the simulations of Scenario 2, but this time accounting for reversibility of the [RANKL/OPG] binding. We introduced a new state variable ϕ_{RO} and added the following reaction-diffusion equation to system (6) of the main text,

$$\frac{\partial \phi_{RO}}{\partial t} = \sigma_{RO} \frac{\partial^2 \phi_{RO}}{\partial x^2} - k_4 \phi_{RO} + k_3 \phi_R \phi_O, \tag{6}$$

where k_4 (units are d^{-1}) is the dissociation rate, and σ_{RO} is the diffusion constant for the complex. We assume that the complex remains membrane-bound, and therefore we set $\sigma_{RO} = \sigma_R$, the diffusion rate

of membrane-bound RANKL (see Section 3). Furthermore, conservation of mass dictates the addition of the term $+k_4\phi_{RO}$ to the RANKL and OPG equations, respectively. Fixing an intermediate background RANKL level of $\phi_R^0 = 2$, we repeated Scenario 2 of the main text for three different values of $k_4 = 0$, $k_4 = 0.1$, and $k_4 = 0.3$, see Figure 2 of SI Text 1. The dissociation of the [RANKL/OPG] complex leads to a slight decrease of the distance traveled by the cutting cone, but it also results in higher osteoclast numbers in the cutting cone. Importantly, the qualitative impact of low to intermediate OPG production on tumor burden remains unaltered (compare to Figure 5C in main text).

2 Numerics

2.1 Boundary Conditions

In [1,2], the simulations were performed on numerical domains that were large enough to avoid interactions between the bone remodeling unit and the domain boundaries. Therefore, Dirichlet boundary conditions in conjunction with spatial finite difference discretisations yielded good results. However, for the current implementation of the model we used periodic boundary conditions. This choice facilitated the use of spectral collocation methods for the discretisation of the Laplacian. In order to make sure that the size of the domain did not affect the simulations, we first solved the equations on the original domain, then doubled the domain size while keeping the mesh size constant, and eventually verified that the relative difference between the two solutions was negligible.

2.2 Fractional Step Method

The model equations of *Scenarios 1-5* share a common feature: they all involve multiple time scales and hence suffer from stiffness, see e.g. [13]. In fact, the reaction and decay rates for the cytokines are of the order of an hour, whereas the chemotactic motion of the remodeling front has a time scale of about a week. If one were to perform a combined time-stepping for all state variables, the stiffness would strongly suggest the use of an implicit method. However, since most terms in the equations are non-linear, an implicit time-stepping would be very expensive. Furthermore the chemotactic term, essentially hyperbolic in nature, is best tackled with an explicit method. These considerations lead us to solve the model equations with a fractional step method as proposed in [14]. Given an evolution equation with advective ($\mathcal{A}$), diffusive ($\mathcal{D}$) and reactive ($\mathcal{R}$) contributions,

$$\partial_t q = \mathcal{A}(q) + \mathcal{D}(q) + \mathcal{R}(q),$$

the fractional step method consists of a sequence of iterative integrations. More precisely, given the value $q^n := q(t_n)$, the method integrates the solution over Δt in three steps:

1. **Advection.** Solve $\partial_t q = \mathcal{A}(q)$ with initial datum q^n over Δt to obtain q^*
2. **Diffusion.** Solve $\partial_t q = \mathcal{D}(q)$ with initial datum q^* over Δt to obtain q^{**}
3. **Reaction.** Solve $\partial_t q = \mathcal{R}(q)$ with initial datum q^{**} over Δt to obtain q^{n+1}.

The main advantage of this approach is that for each contribution one can choose the best suited numerical method. For the **advection step** we used a second order centred difference scheme to discretize the spatial derivatives of the chemotactic term in the osteoclast equation. The time stepping between t and $t + \Delta t$ was performed by means of the `Matlab` built-in adaptive Runge-Kutta 45 solver `ode45`. Regarding the **diffusion step** we followed [14] and implemented TR-BDF2, an implicit Runge-Kutta method of second order. As for the spatial discretization of the Laplacian, we implemented a standard spectral collocation method, see e.g. [15]. Finally, the **reaction step** was integrated with `ode45`, similarly to the advection step. Even though the reaction part is mildly stiff for some *Scenarios*, `ode45` performed well. To ensure that the employed numerical schemes were convergent, we performed convergence studies.

3 Parameter estimation

Similarly to the approach described in Section 4.1 of [2], we distinguish between *fixed* and *free* parameters. *Fixed* parameters are estimated based on experimental findings, whereas the remaining – *a priori* unknown – *free* parameters are determined according to the following *tuning strategy*. While keeping the *fixed* parameters unchanged, the *free* ones are tuned within a reasonable numerical range until the solution matches the following *in vivo* observations: the remodeling front of active osteoclasts stays spatially well-confined and moves across the trabecula at a roughly constant speed of $20 - 40\ \mu m/day$. In the remainder of this section, we implement this strategy and discuss individual parameters. Except for the parameters that vary from scenario to scenario, the values of *fixed* and *free* parameters are summarized in (7) and (8), respectively.

In a first step we consider the osteoclast (u), RANKL (ϕ_R) bone (ρ_B) and tumor (ρ_T) fields of *Scenario 1*, see equation (6) in the main text (ignoring the OPG and PTHrP fields). The parameters α, β and g, corresponding to the internal dynamics of the osteoclast population, have already been determined in [2] and are considered *fixed*. Since RANKL is assumed to be membrane bound, the experimentally unknown effective diffusion rate σ_R should be at least one order of magnitude smaller than the diffusion rates of soluble OPG and PTHrP (whose values are discussed below). Performing simulations with σ_R more than one order of magnitude smaller than σ_O and σ_P leads to numerical instabilities as is to be expected for an advection–dominated reaction–diffusion–advection system. In consequence, we choose σ_R to be one order of magnitude smaller than σ_O and σ_P. If new experimental evidence would reveal that σ_R is much smaller than assumed, our numerics would have to be reviewed. The remaining (*free*) parameters in *Scenario 1*, namely k_1, k_2, λ, ζ and k_B could not be matched to any experimental data and hence we employed the *tuning strategy* outlined above.

For the additional fields appearing in *Scenarios 2–5*, namely OPG and PTHrP, as well as their interactions with the RANKL and bone fields, we have the following *fixed* parameters. First, the diffusion rates of OPG and PTHrP can be estimated from related experimental findings: in [16], effective diffusion rates for Verteporfin in subcutaneous and orthotopic tumours were measured to be 0.88 and 1.59 $\mu m^2\, s^{-1}$ respectively. Assuming that the diffusion rate σ of a molecule of mass M scales as $\sigma \sim M^{-1/3}$, see [17], we can use these rates together with the molecular weights of OPG as a dimer ($\sim$ 120 kDa [18]), PTHrP ($\sim$ 18 kDa, [19]) and Verteporfin ($\sim$ 0.7 kDa, `www.drugbank.ca`) to determine the range of possible effective diffusion rates of OPG and PTHrP in tumour tissue. For OPG we obtain a range of $\sigma_O \sim 1.4 - 2.5 \cdot 10^{-2} mm^2 day^{-1}$ and for PTHrP $\sigma_P \sim 2.6 - 4.7 \cdot 10^{-2} mm^2 day^{-1}$. For the present study, we fix $\sigma_O = 1.6 \cdot 10^{-2}$ and $\sigma_P = 3 \cdot 10^{-2}$. Note that the chosen scaling law $\sigma \sim M^{-1/3}$ applies to globular molecules, but may not be accurate for rod-like molecules. Since OPG and PTHrP have both globular and non-globular domains, a strict classification does not seem possible. Furthermore, we could not find a reliable source for the long and short axes of the molecules (which appear in the scaling relations for rod-like molecules), and hence we used the globular scaling relation to estimate the coefficients. Regarding the half-life of OPG, experimental data range between 10 minutes in rats [20] and 4 days in monkeys [21]. Converted into reaction rates this gives us a ballpark of $k_O \in [0.6, 110]\, day^{-1}$. We decided to settle for an intermediate order of magnitude by choosing $k_O = 10\, day^{-1}$. Even though the half-life of PTHrP is expected to be of the same order of magnitude as the one of OPG, we could not find any corresponding experimental studies. Therefore, we treated k_P together with the remaining parameters τ_O, τ_R, τ_P and k_3 as *free* parameters and used the *tuning strategy*.

In summary, the *fixed parameters* are (we abbreviate *day* by *d*)

$$\begin{array}{llll} \alpha = 9.49\, mm^{-1/2} d^{-1} & \beta = 0.2\, d^{-1} & g = 0.5 & \sigma_R = 0.5 \cdot 10^{-2}\, mm^2 d^{-1} \\ \sigma_O = 1.6 \cdot 10^{-2}\, mm^2 d^{-1} & \sigma_P = 3 \cdot 10^{-2}\, mm^2 d^{-1} & k_O = 10\, d^{-1} & \kappa_R = 1\, d^{-1}, \end{array} \quad (7)$$

and the *free parameters* are

$$\begin{array}{llll} k_1 = 0.3\, d^{-1} & k_2 = 0.05\, pmol\, d^{-1} & \lambda = 13\, pmol\, mm^{-1} & \zeta = 1.3 \cdot 10^{-3}\, mm^3 pmol^{-1} d^{-1} \\ k_B = 3\, d^{-1} & k_3 = 0.1\, mm\, d\, pmol^{-1} & k_P = 4\, d^{-1}. & \end{array} \quad (8)$$

A final note on parameter sensitivity is in order. Given the large number of parameters in the model, a systematic computational sensitivity analysis is not feasible. However, the sensitivity of the various parameters has been investigated in several steps. (A) Parameter sensitivity of the osteoclast-internal dynamics (production, apoptosis, autocrine stimulation) was assessed analytically in [22]. (B) Parameter sensitivity for the underlying spatio-temporal model of bone remodeling was studied by means of scaling arguments in [2]. (C) Sensitivity with respect to the parameters introduced in the current study (e.g. cytokine production rates by tumor, parameters in the PTHrP field) was assessed computationally over a wide range in parameter space.

References

1. Ryser M, Nigam N, Komarova S (2009) Mathematical modeling of spatio-temporal dynamics of a single bone multicellular unit. J Bone Miner Res 24: 860–870.

2. Ryser M, Komarova S, Nigam N (2010) The Cellular Dynamics of Bone Remodeling: A Mathematical Model. SIAM J Appl Math 70: 1899–1921.

3. Jones H, Nakashima T, Sanchez O, Kozieradzki I, Komarova S, et al. (2006) Regulation of cancer cell migration and bone metastasis by RANKL. Nature 440: 692–696.

4. Henriksen K, Karsdal M, Delaissé J, Engsig M (2003) RANKL and vascular endothelial growth factor (VEGF) induce osteoclast chemotaxis through an ERK1/2-dependent mechanism. J Biol Chem 278: 48745.

5. Mosheimer B, Kaneider N, Feistritzer C, Sturn D, Wiedermann C (2004) Expression and function of RANK in human monocyte chemotaxis. Arthritis Rheum 50: 2309–2316.

6. Zhao S, Kato Y, Zhang Y, Harris S, Ahuja S, et al. (2002) MLO-Y4 Osteocyte-Like Cells Support Osteoclast Formation and Activation. J Bone Miner Res 17: 2068–2079.

7. Kurata K, Heino T, Higaki H, Väänänen H (2006) Bone marrow cell differentiation induced by mechanically damaged osteocytes in 3D gel-embedded culture. J Bone Miner Res 21: 616–625.

8. Evans L (2010) Partial differential equations, volume 19 of *Graduate Studies in Mathematics*. American Mathematical Soc.

9. Wei J, Gross M, Jaffe C, Gravlin K, Lahaie M, et al. (1999) Androgen deprivation therapy for prostate cancer results in significant loss of bone density. Urology 54: 607–611.

10. Shapiro C, Manola J, Leboff M (2001) Ovarian failure after adjuvant chemotherapy is associated with rapid bone loss in women with early-stage breast cancer. J Clin Oncol 19: 3306.

11. Navone N, Olive M, Ozen M, Davis R, Troncoso P, et al. (1997) Establishment of two human prostate cancer cell lines derived from a single bone metastasis. Clin Cancer Res 3: 2493–2500.

12. Bru A, Albertos S, Luis Subiza J, Garcia-Asenjo J, Bru I (2003) The universal dynamics of tumor growth. Biophysical Journal 85: 2948–2961.

13. LeVeque R (2007) Finite difference methods for ordinary and partial differential equations. Classics in Applied Mathematics. Society for Industrial and Applied Mathematics.

14. Tyson R, Stern L, LeVeque R (2000) Fractional step methods applied to a chemotaxis model. J Math Biol 41: 455–475.

15. Trefethen L (2000) Spectral methods in MATLAB, volume 10 of *Software, environments, tools*. SIAM.

16. Zhou X, Pogue B, Chen B, Hasan T (2004) Analysis of effective molecular diffusion rates for verteporfin in subcutaneous versus orthotopic dunning prostate tumors. Photochem Photobiol 79: 323–331.

17. Young M, Carroad P, Bell R (1980) Estimation of diffusion coefficients of proteins. Biotechnol Bioeng 22: 947–955.

18. Tsuda E, Goto M, Mochizuki S, Yano K, Kobayashi F, et al. (1997) Isolation of a novel cytokine from human fibroblasts that specifically inhibits osteoclastogenesis* 1,* 2. Biochem Biophys Res Commun 234: 137–142.

19. Moseley J, Kubota M, Diefenbach-Jagger H, Wettenhall R, Kemp B, et al. (1987) Parathyroid hormone-related protein purified from a human lung cancer cell line. Proc Natl Acad Sci USA 84: 5048–5052.

20. Tomoyasu A, Goto M, Fujise N, Mochizuki S, Yasuda H, et al. (1998) Characterization of monomeric and homodimeric forms of osteoclastogenesis inhibitory factor. Biochem Biophys Res Commun 245: 382–387.

21. Kostenuik P (2005) Osteoprotegerin and RANKL regulate bone resorption, density, geometry and strength. Curr Opin Pharmacol 5: 618–625.

22. Komarova S, Smith R, Dixon S, Sims S, Wahl L (2003) Mathematical model predicts a critical role for osteoclast autocrine regulation in the control of bone remodeling. Bone 33: 206–215.
