# Supplementary material for: Osteoprotegerin in Bone Metastases: Mathematical Solution to the Puzzle
Source: PLoS Comput Biol. 2012 Oct 18;8(10):e1002703. doi: 10.1371/journal.pcbi.1002703 (PMC3475686; doi:10.1371/journal.pcbi.1002703)
Supplement: Text S2 — Meta-analysis of gene expression. (PDF) [file pcbi.1002703.s002.pdf]

Supporting information II

"Osteoprotegerin in bone metastases: mathematical solution to the puzzle"

Marc D Ryser[1], Yiding Qu[2] and Svetlana V Komarova[3]
[1]Department of Mathematics and Statistics, McGill University, Montréal, Quebec, Canada,
[2]Department of Anatomy and Cell Biology, McGill University, Montréal, Quebec, Canada,
[3]Faculty of Dentistry and Department of Anatomy and Cell Biology, McGill University, Montréal, Québec, Canada

Meta-analysis for OPG, RANKL and PTHrP gene expression

To assess the patterns of expression of OPG, RANKL and PTHrP in prostate cancer, we used publicly available data (www.oncomine.org) for the expression of these genes in the patient samples from normal prostate tissue, prostate carcinoma, and metastatic prostate carcinoma tissues. Nine studies were included in the analysis to assess the mRNA expression level of OPG (gene name: *TNFRSF11B*), five of these studies were included in the analysis to assess the mRNA expression level of RANKL (Gene name: *TNFRSF11*), and seven of these studies were included in the analysis to assess the mRNA expression level of PTHRP (Gene name: *PTHLH*): (Table 1).

According to the tissue type the data were separated into 3 groups, *normal* prostate, prostate *carcinoma* and *metastatic* prostate carcinoma. *Normal* prostate category included data originally labeled as "Prostate Gland", "Normal Prostate", "Normal Adjacent Prostate", "Benign Prostate" and "Normal Adult Prostate". Prostate *carcinoma* category included data originally labeled as "Prostate Carcinoma Primary Site", "Prostate Adenocarcinoma Primary Site". *Metastatic* prostate carcinoma category comprised all types of metastatic carcinoma; including data originally labeled as "Metastatic Prostate Cancer", "Hormone-Refractory Metastatic Prostate Carcinoma", "Metastatic Prostate Carcinoma, Lymph Node", "Lymph Node Metastasis", "Distant Metastasis - Soft Tissue", "Distant Metastasis – Bone", "Metastasis", "Distant Metastasis", "Distant Metastasis – Liver", "Distant Metastasis - Adrenal Gland", "Distant Metastasis – Kidney", "Distant Metastasis – Lung".

Table 1. Studies used in the meta-analysis.

Oncomine identifier	Reporter ID	Platform	Reference
Lapointe Prostate	OPG: IMAGE: 665356	cDNA	Lapointe J, et al. *Proc Natl Acad Sci U S A 2004;* **101**: 811.
LaTulippe Prostate	OPG: 37611_at<br>PTHRP: 37989_at<br>RANKL: 37611_at	Human Genome U95A-Av2 Array	LaTulippe E, et al. *Cancer Res*. 2002; **62**(15): 4499.
Magee Prostate	OPG: U94332_at<br>PTHRP: M17183_s_at	HumanGeneFL Array	Magee JA, et al. *Cancer Res 2001* **61**, pp. 5692.
Ramaswamy Multi-cancer 2	OPG: U94332_at<br>PTHRP: J03580_s_at	OPG: HumanGeneFL Array<br>PTHRP: Hu35KsubA Array	Ramaswamy S, Ross KN, Lander ES, Golub TR. *Nat Genet.* 2003; 33:49.
Ramaswamy	OPG: U94332_at	OPG: HumanGeneFL	Ramaswamy S, et al.

Multi-cancer	PTHRP: J03580_s_at	Array<br>PTHRP: Hu35KsubA Array	*Proc Natl Acad Sci USA.* 2001; **98**: 15149
Tomlins Prostate	OPG: IMAGE:825287<br>PTHRP: IMAGE:1404774<br>RANKL: IMAGE:825287	20,000 element–spotted cDNA microarray	Tomlins, S. A. et al. Nature Genet. **39**, 41–51 (2007).
Vanaja Prostate	OPG: 204932_at<br>PTHRP: 206300_s_at<br>RANKL: 204932_at	Human Genome U133A Array	Vanaja D, Cheville J, Iturria S, Young C. *Cancer Res 2003;* **63**:3877
Varambally Prostate	OPG: 204932_at<br>PTHRP: 206300_s_at<br>RANKL: 204932_at	Human Genome U133 Plus 2.0 Array	Varambally S, et al.. *Cancer Cell 2005;* **8**: 393.
Yu Prostate	OPG: 35107_at<br>PTHRP: 37989_at<br>RANKL: 35107_at	Human Genome U95A-Av2 Array	Yu YP, et al. *J Clin Oncol.*2004;**22**:2790

1) **Osteoprotegerin (OPG)**

Means and standard deviations (SD) for 9 studies were calculated and the outlier values for each category in each study were identified as being outside the range of mean ± 2 SD. As a result, 5 of 109 values were removed from Lapointe prostate, 2 of 15 from Magee Prostate, 1 of 40 from Vanaja prostate, and 2 of 112 from Yu prostate. The resulting study means are given in Tables 2.

Table 2. Average OPG expression in different studies. Data are means ± SD, with the number of the samples in each category given in the parenthesis. To calculate Study average all the data in the study were combined. To calculate Category total, all data in the category were combined.

OPG	Normal	Carcinoma	Metastatic	Study average
Lapointe	-2.53±0.51 (n=39)	-2.52±0.54 (n=56)	-2.12±0.89 (n=9)	-2.49±0.57 (n=104)
LaTulippe	-3.1±1.6 (n=3)	-4.2±1.8 (n=23)	-3.4±1.8 (n=9)	-3.9±1.8 (n=35)
Magee	-2.8±1.2 (n=4)	-2.54±0.66 (n=7)	-2.18±0.30 (n=2)	-2.55±0.79 (n=13)
Ramaswamy 2		-3.2±2.9 (n=10)	-0.28±0.72 (n=3)	-2.5±2.9 (n=13)
Ramaswamy		-3.2±2.9 (n=10)	-0.20±0.70 (n=4)	-2.3±2.8 (n=14)
Tomlins	0.012±0.73 (n=3)	-0.036±0.50 (n=8)	0.23±0.86 (n=5)	0.06±0.63 (n=16)
Vanaja	-2.53±0.06 (n=8)	-2.52±0.04 (n=26)	-2.47±0.16 (n=5)	-2.52±0.07 (n=39)
Varambally	-1.51±0.58 (n=6)	-1.02±0.57 (n=7)	-0.73±0.92 (n=6)	-1.08±0.74 (n=19)
Yu	-2.47±0.12 (n=22)	-2.44±0.24 (n=65)	-2.45±0.17 (n=23)	-2.45±0.21 (n=110)
Category total	-2.38±0.75 (n=85)	-2.60±1.35 (n=212)	-1.94±1.38 (n=66)	

To assess if the data reported in different studies are compatible for the meta-analysis, first, we compared the means of different studies using 2-way ANOVA, which was performed using Matlab function "anovan()" using the study as the first category and prostate tissue type as a second category. The 2-way ANOVA demonstrated that both study ($p = 0$) and cancer types ($p = 0.0012$) are significantly different. To assess which study may represent an outlier, we performed a Q-test for different studies, however none of them passed the test. We concluded that none of the studies can be classified as an outlier, however, since study averages are significantly different likely due to use of different expression platforms, the studies require further processing before meta-analysis can be performed. Therefore, we next normalized the data a) by dividing each value by the study mean, or b) by subtracting the study mean from each value. The resulting averages and standard deviations are presented in Table 3.

Table 3. Average OPG expression levels with standard deviations in different studies following normalization by division or subtraction.

	Normalized by division		Normalized by subtraction	
Study	Average	Std	Average	Std
Lapointe Prostate	-1	0.23	0	0.57
LaTulippe Prostate	-1	0.46	0	1.81
Magee Prostate	-1	0.31	0	0.79
Ramaswamy Multi-cancer 2	-1	1.14	0	2.87
Ramaswamy Multi-cancer	-1	1.21	0	2.84
Tomlins Prostate	1	10.70	0	0.64
Vanaja Prostate	-1	0.028	0	0.07
Varambally Prostate	-1	0.67	0	0.74
Yu Prostate	-1	0.084	0	0.21

As clear from the Table 3, normalization by division affects the distribution widths. This effect is the largest for Tomlins prostate study, due to its low mean value (0.05), therefore we excluded this study for the subsequent analysis of data normalized by division. Two-way ANOVA for the 8 studies normalized by division demonstrated that following normalization by division, the studies are not different ($p = 0.999$), but the OPG expression in different tissue types is different ($p = 0.0024$). Two-way ANOVA for the 9 studies normalized by subtraction demonstrated that the studies are not different ($p = 0.999$), but the OPG expression in different tissue types is significantly different ($p = 0.006$).

We next performed meta-analysis by combining the values for different tissue types for all studies a) in the form given by Oncomine, or normalized by b) division or c) subtraction. For all data sets, the OPG expression demonstrated significant increase in the samples from *metastatic* carcinoma, compared to *normal* or *carcinoma* samples as assessed by one-way ANOVA using on-line statistics resource Vassar Statistics [http://faculty.vassar.edu/lowry/VassarStats.html]. For the original data set, the OPG expression was found to be significantly different in different tissue types ($p = 0.001$), with Tukey post-test demonstrating that OPG expression is significantly increased in *metastatic* prostate carcinoma

compared to *normal* prostate tissue ($p < 0.05$) or prostate *carcinoma* tissue ($p < 0.01$). For the data set normalized by division, one-way ANOVA gives the significant difference for OPG expression in different tissue types ($p = 0.0006$), with Tukey post-test demonstrating that OPG expression is significantly increased in *metastatic* prostate carcinoma compared to *normal* prostate tissue ($p < 0.01$) or prostate *carcinoma* tissue ($p < 0.01$). For the data set normalized by subtraction, one-way ANOVA gives the significant difference for OPG expression in different tissue types (p = 0.006), with Tukey post-test demonstrating that OPG expression is significantly increased in *metastatic* prostate carcinoma compared to *normal* prostate tissue ($p < 0.05$) or prostate *carcinoma* tissue ($p < 0.01$).

A graph of box plot was created using Matlab function “boxplot()”. The ‘whisker’ parameter of the Matlab boxplot function *w* was set at 1.5, which means approximately 99.3% of the value is covered if the data are normally distributed.

2) **RANKL**

Average RANKL expression levels in different studies are given in Table 4.

Table 4. Average RANKL expression in different studies. Data are means ± SD, with the number of the samples in each category given in the parenthesis. To calculate Study average all the data in the study were combined. To calculate Category total, all data in the category were combined.

OPG	Normal	Carcinoma	Metastatic	Study average
LaTulippe	-3.42±0.44 (n=3)	-2.69±0.72 (n=23)	-1.74±1.31 (n=9)	-2.5±1.0 (n=35)
Tomlins	0.46±0.57 (n=8)	0.76 (n=1)	-1.44±0.84 (n=4)	-0.004±0.96 (n=13)
Vanaja	-2.32±0.05 (n=7)	-2.32±0.06 (n=26)	-2.27±0.11 (n=5)	-2.32±0.06 (n=38)
Varambally	-2.8±1.1 (n=6)	-2.9±1.6 (n=7)	-3.1±2.1 (n=6)	-2.9±1.6 (n=19)
Yu	-1.54±0.16 (n=23)	-1.51±0.20 (n=63)	-1.56±0.20 (n=24)	-1.52±0.19 (n=110)
Category total	-1.6±1.2 (n=47)	-1.97±0.77 (n=120)	-1.9±1.1 (n=48)	

First, 2-way ANOVA was performed on these studies using the study as the first category and prostate tissue type as a second category. The 2-way ANOVA demonstrated that the studies are significantly different ($p = 0$) but the cancer types are not ($p = 0.837$). We performed a Q-test for different studies, however none of them was identified as an outlier. We next normalized the data a) by dividing each value on the study mean, or b) by subtracting the study mean from each value. The resulting averages and standard deviations presented in Table 5.

Table 5. Average RANKL expression levels with standard deviations in different studies following normalization by division or subtraction.

	Normalized by division		Normalized by subtraction	
Study	Average	Std	Average	Std
LaTulippe Prostate	-1	0.39	0	1.0
Varambally Prostate	-1	0.53	0	1.56
Vanaja Prostate	-1	0.028	0	0.06
Tomlins Prostate	-1	249.5	0	0.96
Yu Prostate	-1	0.12	0	0.19

Normalization by division affects the distribution widths, affecting especially strongly Tomlins prostate study, due to its low mean value (-0.004), therefore we excluded this study for the subsequent analysis of data normalized by division. Two-way ANOVA for the 4 studies normalized by division demonstrated that following normalization by division, the studies are not different ($p = 0.998$), and the RANKL expression in different tissue types is not different ($p = 0.353$). We next performed 2-way ANOVA for the 5 studies normalized by subtraction. Following normalization by subtraction, the studies are not different ($p = 0.981$), and the RANKL expression in different tissue types is not significantly different ($p = 0.837$).

3) **PTHrP**

The average PTHrP expression levels in different studies are given by Table 6.

Table 6. Average PTHrP expression in different studies. Data are means ± SD, with the number of the samples in each category given in the parenthesis. To calculate Study average all the data in the study were combined. To calculate Category total, all data in the category were combined.

PTHrP	Normal	Carcinoma	Metastatic	Study average
LaTulippe	-3.3±1.5 (n=3)	-2.8±1.3 (n=22)	-4.0±2.0 (n=9)	-3.2±1.6 (n=34)
Magee P	-5.4±2.8 (n=4)	-2.3±1.1 (n=7)	-7.4±3.1 (n=3)	-4.2±2.9 (n=11)
Ramaswamy 2		-0.3±1.2 (n=10)	-0.94±0.92 (n=4)	-0.5±0.51 (n=13)
Tomlins	0.21±0.61 (n=14)	-0.07±0.91 (n=20)	-0.02±0.57 (n=14)	0.02±0.73 (n=48)
Vanaja	-2.23±0.12 (n=8)	-2.24±0.07 (n=27)	-2.26±0.09 (n=5)	-2.24±0.08 (n=40)
Varambally	-1.9±1.6 (n=6)	-1.18±0.87 (n=7)	-1.32±0.37 (n=6)	-1.44±1.05 (n=19)
Yu	-2.60±0.14 (n=22)	-2.52±0.16 (n=64)	-2.60±0.11 (n=23)	-2.55±0.15 (n=109)
Category total	-2.0±1.8 (n=57)	-2.0±1.2 (n=157)	-2.2±1.9 (n=64)	

First, 2-way ANOVA was performed on these studies using the study as the first category and prostate tissue type as a second category. The 2-way ANOVA demonstrated that both the studies ($p = 0$) and the cancer types ($p = 0.0014$) are significantly different. We performed a Q-test for different studies, however none of them was identified as an outlier. We next normalized the data

a) by dividing each value on the study mean, or b) by subtracting the study mean from each value. The resulting averages and standard deviations presented in Table 7.

Table 7. Average PTHrP expression levels with standard deviations in different studies following normalization by division or subtraction.

	Normalized by division		Normalized by subtraction	
Study	Average	Std	Average	Std
LaTulippe Prostate	-1	0.51	0	1.6
Magee Prostate	-1	0.69	0	2.9
Ramaswamy Multi-cancer	-1	2.17	0	1.1
Tomlins Prostate	1	28.4	0	0.73
Vanaja Prostate	-1	0.036	0	0.08
Varambally Prostate	-1	0.73	0	1.05
Yu Prostate	-1	0.061	0	0.15

Normalization by division affects the distribution widths, affecting especially strongly Tomlins prostate study, due to its low mean value (0.02), therefore we excluded this study for the subsequent analysis of data normalized by division. Two-way ANOVA for the 6 studies normalized by division demonstrated that following normalization by division, the studies are not different ($p = 0.988$), but the PTHrP expression in different tissue types is different ($p = 0.025$). We next performed 2-way ANOVA for the 7 studies normalized by subtraction. Following normalization by subtraction, the studies are not different ($p = 0.998$), and the PTHrP expression in different tissue types is significantly different ($p = 0.002$).

We next performed meta-analysis by combining the values for different tissue types for all studies a) in the form given by Oncomine, or normalized by b) division or c) subtraction using one-way ANOVA. For the original data set, the PTHrP expression was not found to be significantly different in different tissue types (*p = 0.0.58)*. For the data set normalized by division, one-way ANOVA gave the significant difference for PTHrP expression in different tissue types ($p = 0.024$), but Tukey post-test failed to identify significantly different samples. For the data set normalized by subtraction, one-way ANOVA gives the significantly difference for PTHrP expression in different tissue types ($p = 0.003$), with Tukey post-test demonstrating that PTHrP expression is significantly decreased in *metastatic* prostate carcinoma compared to prostate *carcinoma* tissue ($p < 0.01$). Based on these analysis, we concluded that we cannot reliably demonstrate the changed in PTHrP expression depending in the tissue type.

4) **Correlation studies**

The correlation test for the metastatic data of OPG, PTHrP and RANKL was done in Excel for n = 56 samples in which both OPG and PTHrP was measured, n = 44 samples in which both OPG and RANKL was measured, and n = 44 samples in which both RANKL and PTHrP was measured. Significance of correlation was assessed using on-line statistics resource Vassar Statistics [http://faculty.vassar.edu/lowry/VassarStats.html] and found to be $p < 0.01$ for correlation between OPG and PTHrP, $p = 0.49$ for OPG and RANKL correlation and $p = 0.37$ for RANKL and PTHrP correlation.
